# Mediators of socioeconomic differences in overweight and obesity among youth in Ireland and the UK (2011–2021): a systematic review

**DOI:** 10.1186/s12889-022-14004-z

**Published:** 2022-08-20

**Authors:** Frances M. Cronin, Sinead M. Hurley, Thomas Buckley, Delfina Mancebo Guinea Arquez, Naeha Lakshmanan, Alice O’Gorman, Richard Layte, Debbi Stanistreet

**Affiliations:** 1 Department of Public Health and Epidemiology, Royal College of Surgeons in Ireland University of Medicine and Health Sciences, Dublin, Dublin 2 Ireland; 2 School of Medicine, Royal College of Surgeons In Ireland University of Medicine and Health Sciences, Dublin, Dublin 2 Ireland; 3 Department of Sociology, Trinity College Dublin, Dublin, Dublin 2 Ireland

**Keywords:** Socioeconomic inequalities, Childhood obesity, Childhood overweight, Social gradient, Mediator

## Abstract

**Background:**

By 2025, adult obesity prevalence is projected to increase in 44 of 53 of European-region countries. Childhood obesity tracks directly onto adult obesity, and children of low socioeconomic position families are at disproportionately higher risk of being obese compared with their more affluent peers. A previous review of research from developed countries identified factors mediating this relationship. This systematic review updates and extends those findings specifically within the context of Ireland and the United Kingdom.

**Objective:**

The aim of this systematic review is to summarise peer-reviewed research completed in Ireland and the United Kingdom between 2011–2021 examining mediators of socioeconomic differentials in adiposity outcomes for youth.

**Design:**

An electronic search of four databases, Ovid MEDLINE, Embase, Web of Science and EBSCOhost was conducted. Quantitative studies, published in the English language, examining mediators of socioeconomic differentials in adiposity outcomes in youth, and conducted in Ireland and the United Kingdom between 2011–2021 were included. An appraisal of study quality was completed. The systematic review followed Preferred Reporting Items for Systematic Reviews and Meta-Analyses guidelines.

**Results:**

Following screening, a total of 23 papers were eligible for inclusion. Results indicate socioeconomic differentials for Ireland and the United Kingdom follow similar patterns to other developed countries and have similar mediating factors including early life and parent-level factors. However, this review identified additional factors that mediate the relationship, namely access to green space and favorable neighborhood conditions. Identifying these factors present further opportunities for potential interventions and confirm the requirement for tailored and appropriate research and interventions for Ireland and the United Kingdom.

**Conclusion:**

This review identified several modifiable factors that should be considered when planning interventions aimed at reducing socioeconomic differentials in adiposity among youth in Ireland and the United Kingdom. Support was found for interventions to be made as early as possible in an at-risk child’s life, with the prenatal and preschool periods considered the most efficacious. Results were equivocal about the role of physical activity in the risk of childhood overweight and obesity. While multi-country analyses provide excellent overviews, country- or area-specific research may produce more nuanced, and potentially more powerful findings, which can help better inform policy responses and interventions.

**Supplementary Information:**

The online version contains supplementary material available at 10.1186/s12889-022-14004-z.

## Background

As a leading cause of preventable morbidity and mortality globally, obesity (OB, adult Body Mass Index (BMI) $\ge$ 30 kg/m^2^) is now classified as a modern-day health crisis [1], with adverse health and economic implications for individuals and society [2, 3]. A recent report projected that by 2025, OB prevalence would increase in 44 of the 53 World Health Organisation (WHO) European-region countries studied. Of these, Ireland is projected to have the highest, with 43% of the population obese, while the lowest (Italy) is projected to have 13% [4]. Addressing the rise in OB is a recognised priority in the Irish [5] and the United Kingdom (UK) [6] health care systems; however, the development of effective policy responses is dependent on the knowledge of what risk factors are associated with OB, the stage at which those risk factors are most potent, and which interventions are most effective for the at-risk cohort.

A high percentage of adult OB has its roots in childhood, with OB status persisting as the child matures: 55% of obese children will be obese in adolescence, and 80% of those obese in adolescence will remain obese entering adulthood [7]. It is generally recognised that one of the most effective routes to establishing long-term, sustainable change in the OB profile of a population is to address OB in early life [8]. Currently, with 25% of Irish youth [5], and 33% of UK children [6] classified as overweight (OW, BMI 25–30 kg/m^2^) or OB, it is critical that effective interventions be identified to address the child-to-adult patterning of OB [5, 9].

Recently, the prevalence of OB in children of economically-advanced countries has been seen to plateau, but OB continues to rise among children of low socioeconomic position (SEP) families leading to increasing differentials in risk of OB between SEP groups [3, 10–16]. In Ireland and the UK, there is evidence to suggest that differentials in the risk of OB by SEP begin as young as age three, are well established by age five, and widen with age [16–18]. A recent analysis of UK longitudinal data suggests SEP differentials in childhood BMI outcome first became evident in the UK in 2001, since when they have persisted and widened [12].

Understanding what factors might mediate the association between low SEP and adiposity in youth is vital in order to inform policy development. A recent systematic review summarised evidence from research undertaken in Organisation for Economic Co-operation and Development (OECD, with 38 member countries including the United States of America (USA) and Australia) countries of mediators that contribute to differentials in SEP and adiposity among youth. Reporting on over 28 studies that took place between 1990 and 2016, a number of modifiable risk factors were identified, including early life experience (particularly breastfeeding, early weaning, and maternal smoking in pregnancy); child dietary behaviours (particularly consumption of sugar-sweetened beverages and breakfast-eating patterns); child sedentary activity (particularly television viewing and computer use); and maternal BMI [19]. While these findings are informative at an OECD level, there is wide heterogeneity in the culture and living conditions experienced by youth of OECD countries, making the relevance of outcomes in relation to a specific region or country (e.g. Ireland) unclear.

To date, there has been no systematic or scoping review of studies examining the area of SEP differentials in OB outcomes in the youth of Ireland and the UK. This review was undertaken to present an updated and comprehensive review of all existing research published between 2011–2021, reporting on factors that mediate or contribute to the relationship between SEP and adiposity and OB in youth in Ireland and the UK. The aims of this review were to potentially inform future policy discussions, and to identify any research gaps which might require further investigation.

## Methods

The review was conducted and reported according to the Preferred Reporting Items for Systematic Reviews and Meta-Analyses (PRISMA) guidelines [20]. The protocol of this systematic review has been registered and is available on the Open Science Framework [21].

Studies reported in peer-reviewed journals were included if they had employed quantitative methods, were conducted in Ireland and/or the UK, were published in the English language between the years 2011 and 2021, reported on mediators of the association between at least one indicator of SEP and at least one indicator of adiposity, and had a study cohort aged 18 years or under.

Studies employing qualitative methodology were excluded, as was grey literature, studies where analytic methods were not clearly reported, studies conducted among clinical populations, studies employing ethnicity as an indicator of SEP, studies assessing underweight or stunting as an outcome measure, and/or studies assessing birthweight as an outcome measure.

With the aid of an experienced information specialist, the following bibliographic databases were interrogated (with a limitation of a date range of 2011 and August 4^th^ 2021): Ovid MEDLINE, Embase, Web of Science and EBSCOhost. The search strategy was based on that employed by Gebremariam et. al. [19], with the purpose of extending and extrapolating from their earlier review while targeting Ireland and the UK only. The search was conducted on August 5^th^ 2021.

An example of the final search strategy for one of the databases (Ovid MEDLINE®) is presented in Appendix 1.

The articles were reviewed in two phases. For the first level of screening (title and abstract), the database search results were imported into Rayyan [22], a web-based software for managing systematic reviews. Five researchers (TB, NL, SH, DM, AO) worked in independent pairs to screen articles for inclusion or exclusion, based on title and abstract only. Screening was conducted blind, with any discrepancies resolved by discussion with the larger group. Duplicates were identified and removed prior to discussion.

**Fig. 1:**
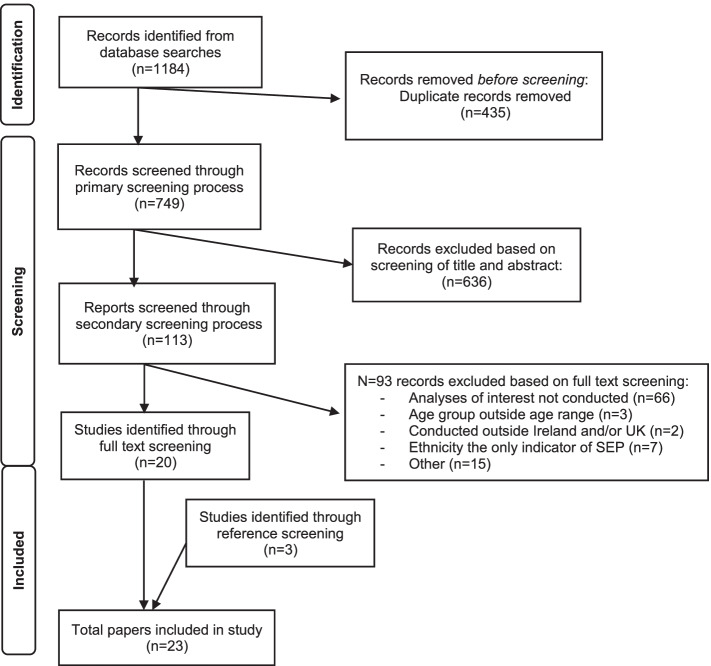
Flowchart indicating steps followed in literature search

For the second level of screening (full text), all papers were transferred to an Excel spreadsheet allowing separate analysis for both included and excluded studies. For included studies, a second review, based on full text, was completed. Again, working in pairs, any discrepancies were resolved by discussion with the larger group, with resulting articles included in the final analysis. Excluded papers were coded for reason of exclusion. Additional papers were identified by examining references of the papers found through the initial search. Screening steps and outcomes are presented in Fig. 1.

All remaining papers underwent data extraction, with information being collated in an Excel spreadsheet based on those items extracted by Gebremariam et. al. [19]. The following items were charted: title; authors; journal; volume; issue; year; pages; type of paper; country conducted; indicator of adiposity; indicator of body weight; indicator of SEP; mediating factors (e.g. child diet, maternal BMI, smoking etc.); time period conducted; population (e.g. infant, child, adolescent, youth); ethnicity; n (% female); methods; mediated relationship (including direction of the association); methods used to assess mediation (name of model used); mediation results; main findings; comments and further work.

A critical appraisal of each journal article was completed using an adapted version of the Liverpool Quality Assessment Tool [23] and the Effective Public Health Practice Project Quality Assessment Tool [24]. Categories of techniques employed for each study were assessed and totalled, generating an overall quality score ranging from ‘strong’ to ‘moderate’ to ‘weak’. Techniques included: Selection Procedures (assessing selection bias and validity of methods); Baseline Assessment (assessing differences between selected groups); Outcome Assessment (assessing dropouts and withdrawals); Analysis (assessing confounding variables and statistical methods); and Impact (assessing the study’s applicability to this review).

## Results

The initial search returned 1184 articles, which reduced to 749 once duplicates were removed. Of these, 636 were excluded following review of title and/or abstract. A full text review took place for 113 articles, following which 93 were excluded. Full data extraction was conducted on 20 articles from the original search and an additional three papers identified by checking reference lists of the included articles. A total of 23 papers were included in the final review [17, 25–46]. See Fig. 1.

Tables 1 and 2 describe the studies included in this review. The majority of the studies used UK data (*n* = 21), while only 10% (*n* = 2) used data from Ireland. Most of the studies were longitudinal in design (*n* = 18), with the remaining (*n* = 5) cross-sectional (Table 1).

**Table 1:** Characteristics of studies included in scoping review

Author (year) and country	Sample Characteristics (n, age (SD), % female)	Study design & instruments	Indicator of body weight (including measurement method and categorisation, descriptives)	Indicator of socioeconomic position	Mediators	Quality Score
Cetateanu et al., 2014, UK [46]	2007–08: *n* = 973,073 and 2008–09: *n* = 1,003,849 and 2009–10: *n* = 1,026,366, age: 4–5 years, 10–11 years	Cross-sectional, data from census, ONS and GIS (NCMP)	Objectively measured wgt and hgt, defining BMI > = 85th and < = 95th percentile as OW and obesity as > 95th percentile using UK90 BMI references	IDACI (measuring relative deprivation including income, employment, education, skills and trainings, health and disability, crime, barriers to housing and services, and living environment), and area SEP	Geography (food environment characteristics: counts of fast food, other unhealthy food, mixed food outlets)	Strong
Goisis et al., 2016, UK [39]	*n* = 11,965, age: 5 years and *n* = 9,384, age: 11 years, 48.4% female	Longitudinal, home visit interviews (PCG) (MCS)	Objectively measured wgt and hgt, defining OW/OB using IOTF criteria; Sample average: 20% OW, 5.8% OB	Family income	Early life (maternal prenatal smoking, breastfeeding duration, weaning); child health behaviours (physical activity, child sedentary behaviour, active play with parent, sleep time, mode of travel to school); screen time (television and computer); child diet (breakfast, fruit and sugar drink consumption); parent-level (maternal BMI)	Moderate
Goisis et al., 2019, UK [31]	*n* = 11,331, age: 7 years, 49.5% female	Longitudinal, home visit interviews (PCG) (MCS)	Objectively measured wgt and hgt, categorized into non-overweight, OW/OB using IOTF cut-offs	Family income	Ethnicity; early life (maternal prenatal smoking, breastfeeding duration, weaning); child health behaviours (sport/exercise, active playing with parent, regular bedtime); Screen time/media exposure (television and computer use); child diet (breakfast, fruit, sugar drinks); parent-level (maternal overweight/obese at time of survey; parenting: meals eaten with parent)	Moderate
Laverty et al., 2021, UK [40]	*n* = 8,432, age: 7, 11 and 14 years, 51.3% female	Longitudinal, home visit interviews (PCG and child) (MCS)	Objectively measured wgt and hgt, used to calculate BMI and % BF	Family income, occupational social class	Child health behaviour (mode of travel to school)	Strong
Layte et al., 2014, Ireland [17]	*n* = 9,057, age: birth, 9 months and 3 years, 49% female	Longitudinal, home visit interviews (PCG) and health records (GUI)	Birthweight: taken from health professions birth records converted to z scores. Age 9 months: objectively measured wgt converted to z scores. Age 3: objectively measured wgt and hgt, categorized into OW/OB using IOTF cut-offs	Household social class (Irish Central Statistics office)	Early life (maternal prenatal smoking and alcohol consumption, duration of breastfeeding, weaning); child diet (dietary quality index); screen time/media exposure (television and DVD use)	Moderate
Lu et al., 2020, UK [43]	*n* = 15,996, age: 3, 5, 7, 11 and 14 years, 48.3% female	Longitudinal, home visit interviews (PCG) (MCS)	Objectively measured wgt and hgt, defining OB and OB using both IOTF and WHO criteria; OW & OB: 28.5% (IOTF); 35% (WHO)	Maternal education, family income	Ethnicity	Moderate
Martinson et al., 2012, UK and USA [37]	US sample: FFS, *n* = 2,930, age 1, 3, 5 and 9 years UK sample: MCS, *n* = 6,816, age 3, 5, 7 and 9 years	US: Longitudinal, parental hospital and home visit interviews. (FFS) UK: Longitudinal, home visit interviews (PCG) (MCS)	US sample: BMI calculated from wgt and hgt at ages 3 and 9 UK sample: Objectively measured wgt and hgt, BMI calculated from wgt and hgt at ages 3 and 7; BMI categorised using CDC guidelines with 85th percentile designating OW	Maternal education, family income	Ethnicity; Parent-level factors (age mother immigrated (under/over 18 years))	Moderate
Massion et al., 2016, UK [25]	*n* = 11,764, age: 11 years, 48% female	Longitudinal, home visit interviews (PCG) (MCS)	Objectively measured wgt and hgt, defining OW/OB using IOTF criteria; 28.8% OW at age 11	Maternal education	Early life factors (maternal pre-pregnancy weight, maternal prenatal smoking, BW, caesarean delivery, breastfeeding duration, weaning)	Strong
Mireku et al., 2020, UK [45]	*n* = 11,714, age: 14 years, 47.6% female	Longitudinal, home visit interviews (PCG) (MCS)	Objectively measured wgt and hgt. BMI used to classify OW and OB using IOTF cut-offs. 8.0% OB, 27.2% OW	Area of deprivation	Household-level (income (equivalised))	Moderate
Noonan et al., 2016, UK [41]	*n* = 194, age: 9–10 years, 55.1% female	Cross-sectional, data from NSPD, in-school interviews (child), parental questionnaires (via school)	Objectively measured wgt and hgt, used to calculate BMI and BMI z-scores. Normal weight and OW/OB defined using IOFT. 26% OW/OB	IMD (family income, employment, health education, housing, environment, crime)	Geography (home and neighbourhood environments (including crime and aesthetics)); child health behaviour (physical activity); parent-level (child bedroom TV)	Moderate
Noonan, 2018, UK [28]	*n* = 10,736, age: 9 months-14 years, 49.5% female	Longitudinal, home visit interviews (PCG and child) (MCS)	Objectively measured wgt and hgt, categorized into non-overweight, OW/OB using IOTF cut-offs; OW 26.6%, OB 7.4%	Family income	Child diet (fruit, veg, sugary drink, and fast food consumption)	Moderate
Noonan et al., 2018, UK [30]	*n* = 3,717, age: 7 years, 51% female	Longitudinal, home visit interviews (PCG), physical activity assessment (MCS)	Objectively measured wgt and hgt, categorized into normal OW OB using IOTF criteria; 17% OW, 14% OB	Maternal education and area deprivation	Child health behaviours (physical activity)	Moderate
Oude Groeniger et al., 2020, UK [27]	*n* = 11,413, age: 7–14 years, 47% female	Longitudinal, home visit interviews (PCG) (MCS)	Objectively measured wgt and hgt, defining OB using IOTF criteria; 8% OB at age 14	Maternal education	Screen time/media exposure (television viewing and computer use)	Strong
Parkes et al., 2016, UK [34]	*n* = 2,957, age: 46, 70 and 94 months, 48.4% female	Longitudinal, in-home interviews (PCG) (GUS)	Objectively measured wgt and hgt, at 46, 70 and 94 months used to derive standardised BMI z-scores using UK90 British growth reference data	Maternal education	Parent-level (parenting: main meal while watching TV, meals eaten in non-dining/food preparation area (e.g. bedroom), child bedroom TV); Child diet (skip breakfast, fruit, veg, crisps, sugar drinks, sweets, and chocolate consumption)	Strong
Samani-Radia et al., 2011, UK [29]	*n* = 2,298, age: 5–14 years, 45.6% female	Cross-sectional, in-school surveys, LEA data	Objectively measured wgt and hgt, categorized into non-overweight, OW/OB using IOTF cut-offs and % BF cut-offs using UK90 growth reference data categorizing overfat and obese	Environment (poorer urban/inner city London area with a high density of social housing) and income characteristics defined at school-level (% of children receiving free school meals)	Child height	Moderate
Schalkwijk et al., 2017, UK [38]	*n* = 6,467, age: 9 months, 3, 5 and 7 years, 49.7% female	Longitudinal, home visit interviews (PCG) observational assessment (interviewer) (MCS)	Objectively measured wgt and hgt, defining OB using IOTF criteria; defining normal, 19.9% OW/OB at 7 years	Parental education, family income	Geography (greenspace, access to garden, condition of neighbourhood)	Strong
Silverwood et al., 2016, UK [36]	*n* = 16,628, age: 6–9 weeks, 21–24 and 39–42 months, and 48 months, 48.4% female	Longitudinal, census and SIMD data, child health records (CHSP Pre-School). (SLS)	Length/height, weight and age derived from CHSP pre-school records at 6–8 weeks, 8–9 weeks, 21–24 months, 39–42 months and 48 months. Predicted BMI at age 4.5 years derived from predicted hgt and wgt values with OW at age 4.5 defined using Cole (2000) standard definition	Maternal education, Scottish IMD, family income	Early life (BW)	Moderate
Straatmann et al., 2020, UK [26]	*n* = 6,306, age: 14 years	Longitudinal, home visit interviews (PCG) (MCS)	Objectively measured wgt and hgt, defining OW/OB using IOTF criteria; 24.6% OW/OB	Maternal education	ACE (verbal and physical maltreatment, parental divorce, drug use, alcohol use, maternal mental illness, domestic violence)	Strong
Strugnell et al., 2020, UK [44]	*n* = 2.35 million, age: 4–5 years, 10–11 years, 49% female	Cross-sectional, school records (NCMP)	Objectively measured wgt and hgt. IOTF growth reference used to classify OW and OB	IDACI (measuring relative deprivation including income, employment, education, skills and trainings, health and disability, crime, barriers to housing and services, and living environment)	Ethnicity	Weak
Stuart et al., 2016, UK [35]	*n* = 9,699, age: 3, 5, 7, 11 years, 50.7% female	Longitudinal, home visit interviews (PCG) (MCS)	Objectively measure wgt and hgt, categorized into OW/OB using IOTF cut-offs	Parental income, parental education, persistent poverty indicator	Early life (maternal prenatal smoking, breastfeeding (never), low BW, high BW)	Strong
Townsend et al., 2011, UK [32]	*n* = 396,171, age: 4–5 years, 48% female and *n* = 392,344, age: 10–11 years, 48% female	Longitudinal, data from NCMP, CWI scores via the DCLG, FSM via school census data from DCSF (NCMP)	Objectively measured wgt and hgt, resulting in z-scores using UK90 growth reference (Cole 1995, 1998)	CWI (a composite score of seven domains: material well-being, health, education, crime, housing, environment, children in need)	School-level deprivation: FSM (% of children receiving free school meals)	Strong
Walsh et al., 2015, Ireland [42]	*n* = 8,599, age: 9 years, 45.1% female	Cross-sectional, home visit interviews (PCG and child) (GUI)	Objectively measured wgt and hgt, defining OB and OW/OB using IOTF cut-offs. 5.3% OB, 24.1% OW/OB	Family income	Geography (urban/rural, proximity to recreational facilities); household-level (home owner); parent-level (age of parents, parent BMI, current smoker, child bedroom media); early life (maternal prenatal smoking and alcohol consumption, breastfed (ever), BW); child health behaviour (frequency of exercise, hospital nights, doctor visits); screen time (TV, computer and video games); child diet (sugar drinks, crisps, chips, junk food)	Strong
Wijlaars et al., 2011, UK [33]	*n* = 2,394, age: birth-3 months, 50.5% female	Longitudinal, questionnaire (PCG), child health records (Gemini study)	Health professions record of infant weight used to calculate weight standard deviation scores at birth and 3 months based on UK90 growth reference data	NS-SEC (based on occupation, maternal education qualifications)	Early life (maternal prenatal smoking, breastfeeding duration, weaning); parent-level (BMI)	Moderate

Abbreviations: *ACE* Adverse Childhood Experience, *ALSPAC* Avon Longitudinal Study of Parents and Children (UK), *BF* Body fat, *BMI* Body Mass Index, *BW* Birth weight, *CI* Confidence Interval**,** *CHSP* Pre-School Child Health System Programme Pre-School (UK), *CWI* Child Wellbeing Index (UK), *DCLG* Department of Communities and Local Government (UK), *DCSF* Department for Children, Schools and Families (UK), *FFS* Fragile Families and Child Wellbeing Study (US), *FSM* Free School Meals, *GIS* Geographic Information System (UK), *GUI* Growing Up in Ireland (Ireland), *GUS* Growing up in Scotland (UK), *Hgt *height, *HSE* Health Survey for England (UK), *IDACI* Income Deprivation affecting Children Index (UK), *IMD* Index of Multiple Deprivation (UK), *IOTF* International Obesity Task Force, *LEA* Local Education Authority (UK), *MCS* Millennium Cohort Study (UK), *NCMP* National Child Measurement Programme (UK), *NS-SEC* National Statistics Socioeconomic Class index (UK), *NSPD* National Statistics Postcode Directory (UK), *OB* Obese, *OECD* Organisation for Economic Co-operation and Development, *ONS* Office for National Statistics (UK), *OW* Overweight, *PCG* Primary Care Giver, *SD* Standard Deviation, *SEP* Socioeconomic position**,** *SIMD* Scottish Index of Multiple Deprivation (UK), *SLS* Scottish Longitudinal Study (UK)**,** *TV* Television, *Wgt* weight

**Table 2:** Factors mediating the association between socioeconomic position and adiposity in youth in Ireland and the UK

Study	Mediated relationship (direction of the association)		Method used to Assess Mediation [name of model used]	Mediation Results*	
Cetateanu & Jones [46]	Association btw deprivation and (a) OB ( +) and (b) OW/OB ( +) for: (1) 4–5 year olds (-) (2) 10–11 year olds (-)		Preacher and Hayes indirect effect method	(1) No mediating effect in the 4–5 year old group (2) For the older cohort, availability of fast food outlets and other types of unhealthy food outlets partially mediated the association btw deprivation and OB and OW/OB by between 1 and 2%. No mediation was found for the availability of mixed food outlets	
Goisis et al. [39]	Association btw family income and risk of: (1) OB at age 5 (-) (2) OW at age 11 (-) (3) OB at age 11 (0) (4) Upward movement across weight categories from age 5 to age 11 (-)		Assessment of attenuation/reduction of regression coefficients upon inclusion of mediators	(1 and 4) Physical activity, TV use, bedtime, fruit intake, sweet drink intake and maternal BMI skipping breakfast did most to attenuate inequalities. Other factors including maternal smoking during pregnancy, breastfeeding duration and time of weaning also played a role in mediation (2 and 3) Fruit, sweet drink, and breakfast intake did most to attenuate inequalities, with other factors (see 1 and 4) playing a smaller role	
Goisis et al. [31]	Association btw family income and OW/OB		Logistic regression models	Poorer White children are at higher risk of OW/OB than higher-income White children (RRR 1.13; 95% CI: 1.02 to 1.25). This SEP differential is reversed for children from Black Caribbean/African backgrounds and non-existent for Indian and Pakistani/Bangladeshi backgrounds. In contrast to White children, lower income children from all other ethnic backgrounds are less likely to be OW/OB at age 7 than their more advantaged counterparts	
Laverty et al. [40]	Association btw household income group and occupational social class with: (1) BMI (2) % BF		Longitudinal (panel) regression models	(1) Switching to active travel was associated with a − 0.32 kg/m^2^ BMI (95% CI − 0.58 to − 0.06) among those in the lowest household income group compared with a -0.11 kg/m^2^ among the highest group (-0.24 to 0.03) (2) Switching to active travel was associated with a − 0.71% BF (95% CI − 1.47% to 0.05%) among the lowest household income group compared with a -0.55% BF (-1.01 to -0.09%) among those in the highest income group	
Layte et al. [17]	Association btw social class (baseline professional class) and: (1) rapid growth from birth to 9 months (2) rapid grow from 9 months to 3 years (3) rapid OB at 3 years		Assessment of attenuation/reduction of regression coefficients upon inclusion of mediators	(1) Breastfeeding and age at weaning most important for non-manual class. Antenatal smoking and alcohol consumption most important for manual and unclassified classes. The model with all mediators reduced coefficients by an average of 76% (2) Child diet, TV viewing and maternal BMI led to highest reductions in all classes. Lower maternal BMI and lower levels of TV viewing mediated lower odds of rapid weight gain (3) Child diet, TV viewing and maternal BMI led to highest reductions in coefficients in all classes. All mediator groups had some contribution	
Lu et al. [43]	(1) Association btw poverty and higher BMI in children (2) Association btw maternal education and higher BMI in children		Mixed-effects fractional polynomial and multinomial regression modelling	(1) Poverty was associated with higher BMI in children of White and South Asian origins, widening with age to 0.75 kg/m^2^ (95% CI, 0.59–0.91) and 0.77 kg/m^2^ (0.26–1.27) at 14 years for the White and South Asian groups, respectively. A reverse income-BMI association in children of Black (African-Caribbean) origin was found with the poverty group having a lower BMI (− 0.37 kg/m^2^ [− 0.71 to -0.04] at 5 years and − 0.95 kg/m^2^ [− 1.79 to − 0.11] at 14 years (2) Similar patterns (see (1)) presented with maternal education and obesity at 14 years	
Martinson et al. [37]	Association btw SEP and child OW		Multivariate logistic regression models	Low SEP children with non-White native- and foreign-born mothers are at lower risk of OW. Low SEP children with white immigrant mothers are at an increased risk of OW	
Massion et al. [25]	Association btw maternal education and childhood OW at age 11		Assessment of attenuation/reduction of RR on inclusion of mediators (Barron and Kenny)	Early life risk factors (maternal pre-pregnancy OW, maternal smoking during pregnancy) reduced the RR from 1.72 (95%CI 1.48 to 2.01) to 1.47 (1.26–1.71)	
Mireku & Rodriguez [45]	Association btw deprivation and (a) OW, OB and (b) %BF		Linear regression models and log-binomial models	When stratified by geographic-level deprivation, the family income gradient in the risk of OB for moderately affluent (2nd, 3rd or 4th quintile deprivation) neighbourhoods was non-significant. However, family income gradient in the risk of OW/OB persisted for the most (RR 5.5 (95% CI 1.0–17.2, p < 0.05) and least (2.4 (1.0–5.8, p < 0.05) deprived quintiles of geographic-level deprivation	
Noonan et al. [41]	Association btw area deprivation and child BMI and waist circumference	Linear regression analyses			A significant inverse association was seen between neighbourhood aesthetics and high deprivation group's BMI (β = − 0.29, *p* < = 0.01) and waist circumferences (β = − 0.27, *p* < = 0.01)
Noonan [28]	Association btw poverty and childhood OW/OB		Adjusted logistic and multinomial logistic regression analyses	Adolescents living in poverty compared to those not living in poverty reported more frequent consumption of sweetened drinks and fast food, and less frequent consumption of fruits and vegetables (OR = 1.92–3.61; *p* < 0.001). Difference in weight status and dietary intake outcomes for girls in poverty were greater (OR = 1.55–3.62; *p* < 0.001) compared to boys (OR = 1.39–3.60; *p* < 0.001)	
Noonan & Fairclough [30]	(1) Association btw individual-level SEP (maternal education) and childhood OW/OB (2) Association btw area-level SEP and childhood OW/OB		Adjusted linear and multinomial logistic regression analysis	Individual-level and area-level SEP were independently related to OW/OB. Higher rates of OW/OB among deprived children were not due to physical inactivity	
Oude Groeniger et al. [27]	Association between maternal education and childhood OB		Causal mediation analysis	At age 14, between 13 and 18% of relative inequalities in childhood OB were reduced if maternal education differences in screen media exposure at age 7 and 11 were eliminated	
Parkes et al. [34]	Association btw maternal education and child BMI trajectory slope (-)		Path analysis	Indirect effect of SEP via all mediators (0.16) comprised 89% of the total effect of SEP level on BMI outcome. Pathway to BMI slope from maternal education through parenting (informal meal setting) and then unhealthy diet accounted for 68% of the significant indirect pathways. The main indirect pathway involving parenting was via the effect of child bedroom TV, with smaller effects of informal meal setting and less positive mealtime interaction. An effect of unhealthy diet (which in turn affected BMI) which did not got through parenting was also seen	
Samani-Radia & McCarthy [29]	Association btw geographical location deprivation, family income and childhood OB and % BF		Chi-square tests	Children from lower incomes were significantly shorter, heavier and higher % BF, with a higher BMI for their age compared with those from a higher income background. A shorter height-for-age of the ‘lower income’ group children may contribute to the income group divisions	
Schalkwijk [38]	Association btw parental education, family income and childhood OW/OB		Logistic regression models	Among low SEP households, lack of garden access and less green space was associated with OW/OB; among higher SEP, poor neighbourhood condition influenced the probability of OW and OB: OR, 95% CI 1.38 (1.12–1.70), 1.38 (1.21–1.70) respectively	
Silverwood et al. [36]	Association btw maternal education, area deprivation and weekly household income with OW/OB		Traditional (Baron & Kenny) and counterfactual-based mediation analyses (bootstrapping to assess significance)	Higher BW in low SEP is associated with increased inequalities in OW	
Straatmann et al. [26]	Association btw maternal education and OW/OB at age 14		Counterfactual mediation analyses	For OW/OB, 19% of the total effect of socioeconomic conditions was mediated through all ACEs investigated	
Strugnell et al. [44]	Association btw income deprivation and childhood OW/OB (1) 4–5 year olds (2) 10–11 year olds		Multivariable logistic regression models	(1 and 2) Ethnicity has an independent influence on OW/OB for both groups (4–5 and 10–11 year olds), with the distribution between most and least advantaged widening for most ethnic groups between ages 4–5 and 10–11 years (2) For the 10–11 year olds, SEP differentials were found to differ by sex and by ethnicity with the largest disparity reported for White children, and the smallest seen in Black African children. Comparing boys in the least deprived and most deprived groups, the difference was 12% among White British boys and 18% for Any Other White Backgrounds, compared with 11% for Indian boys, 5% for Pakistani boys and 2% for Black African boys	
Stuart & Panico [35]	Association btw parental income, parental education and a persistent poverty indicator with (1) OW (2) OB		Multinomial logistic regression models	High BW (RRR: 2.16, *p* < 0.05), not being breastfed (RRR: 1.33, *p* < 0.05) and mother smoking during pregnancy (RRR: 1.96, *p* < 0.001) mediated some educational gradient (but not income gradient) between the OB and normal weight group. SEP does not uniformly impact BMI trajectories, and different indicators of disadvantage capture different trajectories. For SEP inequalities, the OW group was mostly characterized by low parental income, whereas the OB group was mostly characterized by low parental education	
Townsend et al. [32]	Association btw area deprivation and BMI		Cross-classified multilevel regression models	Longer time spent in school with a high percentage of children receiving FSM (poorer schools) affected the association found between BMI and low SEP. Deprivation explains a greater proportion of the variance in BMI for older compared with younger children, perhaps reflecting the impact of deprivation as children age, highlighting the widening of health inequalities through childhood	
Walsh & Cullinan [42]	(1) Association btw household income and childhood OB and OW/OB (2) Inequality in OW/OB (based on concentration indices)		Prediction of inequality gradient using regression	Parental occupation and education contributed to OB (41.16%) and OW/OB (44.18%) inequalities; parental health (maternal BMI and maternal smoking during pregnancy) contributed OB (3.7%) and OW/OB (84.1%) inequalities. Child variables had a low impact on observed inequalities—mainly via TV viewings and bedroom TV	
Wijlaars et al. [33]	(1) Association btw parental occupation (NS-SEC Index) and 3-month weight (-), weight gain btw birth and 3 months (-) and rapid weight gain (-) (2) Association btw maternal education and 3-month weight (-), weight gain between birth and 3 months (-) and rapid weight gain (-)		Assessment of attenuation/reduction of regression coefficients upon inclusion of mediators (bootstrapping to assess significance)	(1) Breastfeeding duration attenuated the association btw parental occupation and: 3-month weight by 68%; weight gain by 62%; and odds of rapid growth by 53% (2) Breastfeeding duration attenuated the association btw maternal education and: 3-month weight by 88%; weight gain by 82% and odds of rapid growth by 64%. No mediating effect was found for smoking during pregnancy, maternal and paternal BMI	

Abbreviations: *ACE* Adverse Childhood Event**,** *BF* Body fat**,** *BMI* Body Mass Index**,** *BW* Birthweight**,** *Btw* between**,** *CI* Confidence Interval**,** *FSM* Free school meals, *NS-SEC* Index National Statistics Socioeconomic Class index (UK), *OB* Obesity**,** *OR* Odds Ratio**,** *OW* Overweight**,** *RR* Relative Risk**,** *RRR* Relative Risk Ratio**,** *SEP* Socioeconomic position**,** *TV* Television

As children develop and grow, BMI changes considerably, necessitating the use of centile curves with variable cut-offs to denote OW and/or OB – each calculated by sex for different ages. Cut-off values are available using the British 1990 reference (UK90) published by the Child Growth Foundation [47, 48–51], the US Centers for Disease Control (CDC) charts [52], the International Obesity Task Force (IOTF) [53, 54], and the World Health Organisation (WHO) BMI-for-age cut-offs [55]. IOTF cut-off points were used to define OW and OB from BMI measures in the majority of studies (n = 16) [17, 25–31, 35, 38, 39, 41–45], with n = 3 using UK90 cut-off points [34, 37, 46], and one using both methods [29]. Both IOTF and WHO criteria were used in one study [43] while CDC cut-offs (with no references given) were used for one study [37]. One study did not employ cut-offs [40]. Table 1 also summarises indicators of SEP used: single indicators of SEP were employed in 14 studies [17, 25–28, 31–34, 39, 41, 42, 44, 45]. The remaining nine studies used a combination of measures to identify SEP [28, 29, 35–38, 40, 43, 46].

Table 2 details potential mediators examined and combinations of mediators used. Potential mediators of socioeconomic differences in adiposity were broken down into categories: early life factors *n* = 9; child screen time *n* = 6; child diet *n* = 6; parent-level factors *n* = 6; child health and behaviours *n* = 6; geographical factors *n* = 4; household-level factors *n* = 3; ethnicity *n* = 4; adverse childhood events *n* = 1; child height *n* = 1; and school-level factors *n* = 1. Table 3 provides a summary of variables in each category.

**Table 3:** Breakdown of categories of potential mediators of differentials in SEP adiposity outcome

Early life	Maternal pre-pregnancy weight, maternal prenatal smoking, maternal prenatal drinking, birthweight, delivery type, breastfeeding duration (and never), time of weaning
Child screen time	Television viewing, computer use, video gaming, DVD use
Child diet	Consumption of fruit, vegetable, sugar drink, crisps, chips, junk food, fast foods, sweets, chocolate, breakfast, dietary quality index
Parent-level factors	Maternal BMI, parental BMI, parent weight status, age of parents, mother age of immigration, parent current smoker, parenting style (main meal while watching television, meals eaten in non-food area, child bedroom media)
Child health and behaviours	Physical activity, sport/exercise, sedentary behaviour, sleep time, active play with parent, mode of travel to school, hospital nights, doctor visits
Child-level factor	Child height
Geographical factors	Greenspace, access to garden, condition of neighbourhood, home and neighbourhood environment, crime, aesthetics, urban/rural, proximity to recreational facilities, counts of fast/other unhealthy/mixed food outlets, proximity of fast food outlets
Household-level factors	One/two adult households, home owner, family income, perceived effect of economic recession, financial difficulty, place of birth, race/ethnic group
ACE	Verbal and physical maltreatment, parental divorce, parental drug use, alcohol use, maternal mental illness, domestic violence, number of residential moves, parent death, parent job loss
School-level factors	Deprivation based on the percentage of children receiving free school meals

Abbreviations: *ACEs* Adverse Childhood Event**,** *BMI* Body Mass Index**,** *SEP* Socioeconomic Position

### Mediators of the association between socioeconomic position and adiposity

#### Mediators of the association between deprivation scores and adiposity

Of the fourteen (61%) studies using single indicators of SEP, deprivation scores were used in five. The positive association between deprivation scores and prevalence of childhood OW, OB, and/or OW and OB, was mediated by: parent-level factors [41]; child health behaviours [41]; geography [41, 46]; household-level factors [45]; ethnicity [44]; and school-level factors [32]. Deprivation-based SEP differentials differed by sex and were reported to widen between the ages of four to five years and 10–11 years for most ethnic groups (the largest disparity seen in White children and the smallest seen in Black African children).

#### Mediators of the association between maternal education and adiposity

The association between maternal education and increased risk of adiposity was mediated by early life factors of maternal pre-pregnancy OW and maternal smoking during pregnancy [25]; Adverse Childhood Events (ACE) in the first five years of life [26]; screen time, with five or more hours a day of screen time being associated with a 1.7 fold increased risk of OB [27]; and parenting-level factors, with bedroom TV availability identified as the most important parenting pathway followed by informal meal settings [34].

#### Mediators of the association between parental/family level factors and adiposity

For 9-year old children in Ireland, the majority of SEP inequalities in childhood OB were explained by parental health and maternal BMI, which when added to other parental health traits (such as smoking and drinking habits) was as large, or a larger contributor to OB/OW inequalities than any other group of factors [42]. A relatively low impact for child-level variables (including media use, bedroom TV, and fizzy drink consumption) was found [42]. For UK children and preteens (aged five and 11), childhood physical activity and diet were reported to be important in explaining the differentials in OW/OB outcomes [39]. For UK adolescents, dietary intake mediated the association found between SEP and OW/OB, particularly for girls [28].

Ethnicity also mediated the association, with White children from poor backgrounds shown to be at greater risk of OW/OB than White children from wealthier families. This effect was reversed for Black African/Caribbean children and was non-existent for children of Indian and Pakistani/Bangladeshi origin [31]. Within this, early life factors (including maternal smoking during pregnancy and duration of breastfeeding) and maternal health behaviours (including BMI, breakfast-eating habits, and level of physical activity) explained differences in the White ethnic group but had no effect on the Black Caribbean and African groups [31].

#### Mediators of the association between NS-SEC- UK/Ireland and adiposity

Using national statistics as SEP indicators, for studies in Ireland, the relationship was mediated by early childhood factors: maternal and antenatal lifestyle behaviours and screen time. Child diet and screen time had a greater effect than either early nutrition or maternal prenatal behaviours [17]. In the UK, the association remained significant after including early life factors (smoking during pregnancy) and parental BMI in the models and was attenuated by 68% when breastfeeding was included [33].

#### Mediators of the association between school-level deprivation and adiposity

One study defined SEP using school-level and neighbourhood characteristics. Here, the association was found to be mediated by child height, with a shorter height-for-age of the ‘lower income’ group children contributing to the income group differentials. It should be noted that this study was “restricted to Caucasian children” [29].

#### Mediators of the association between multiple socioeconomic markers and adiposity

Trajectories of BMI varied by ethnicity, with poorer White children heavier than their non-poor peers, and the reverse seen for children of Black African-Caribbean origin: the poverty group had a lower mean BMI than the non-poor group [43]. Furthermore, low SEP children of non-White native and foreign-born mothers were found to be at lower risk of OW compared to children of White mothers. Children born to White immigrant mothers were associated with an increase in the risk of OW [37].

Lack of garden access and less green space increased the risk of OW/OB in lower educated households, while poor neighborhood conditions among higher educated households increased the probability of OW and OB [38]. It is of note that recommended physical activity levels were achieved by the low SEP group, suggesting that higher rates of OW and central OB among deprived children are not due to physical inactivity [30].

Switching to active travel had a greater reduction in both BMI and percentage body fat for those in the lowest household income group compared with those in the highest income group. Similarly, switching to active travel was associated with a greater reduction in lower body fat among those in the economically inactive NS-SEC group, compared with those in the managerial/professional NS-SEC group [40].

While higher birthweight in the more disadvantaged groups increased the SEP differentials found in OW/OB outcome at age four and a half [36], in the youngest age-group, outcomes differed by definition of SEP. Disadvantaged OW children were mostly characterised by low parental income, while disadvantaged OB children were mostly characterised by parental education. Factors in infancy and pregnancy did not mediate the relationship between lower income and OW, although high birthweight, maternal smoking during pregnancy, and not being breastfed mediated some of the educational differentials [35].

### Summary of mediation findings

Table 4 presents studies by SEP indicator and factors examined. Factors assessed in three or more studies, with mediating effects documented in 60–100% of those studies were as follows: early life (seven [17, 25, 31, 33, 36, 39, 42] of nine studies); child diet (five [17, 28, 31, 34, 42] of six studies); parent-level factors (five [31, 33, 34, 41, 42] of six studies); child health behaviours (four [28, 40–42] of six studies); screen time (three [17, 27, 42] of six studies); geography (four [38, 41, 42, 46] of four studies); ethnicity (four [31, 37, 43, 44] of four studies); and household-level factors (three [37, 42, 45] of three studies). For school-level deprivation [32], childhood ACE [26], and child height [29], each was found to have a mediating effect; however, as each was examined in one study only, there is insufficient evidence to draw definitive conclusions.

**Table 4:** Studies by SEP indicator and factors examined

	Maternal/paternal education	Family/Household income	National statistic	Deprivation score	Neighbourhood/School SEP	Multiple measures
Early life	Massion et al. 2016 [25]	Goisis, Sacker, and Kelly 2016; Goisis, Martinson, and Sigle 2019; Noonan 2018; Walsh and Cullinan 2015 [28, 31, 39, 42]	Layte et al. 2014; Wijlaars et al. 2011 [17, 33]	-	-	Silverwood et al. 2016; Stuart and Panico 2016 [35, 36]
Screen time	Oude Groeniger, De Koster, and Van Der Waal 2020 [27]	Goisis, Sacker, and Kelly 2016; Goisis, Martinson, and Sigle 2019; Walsh and Cullinan 2015 [31, 39, 42]	Layte et al. 2014 [17]	Noonan et al. 2016 [41]	-	-
Child diet	Parkes et al. 2016 [34]	Goisis, Sacker, and Kelly 2016; Goisis, Martinson, and Sigle 2019; Noonan 2018; Walsh and Cullinan 2015 [28, 31, 39, 42]	Layte et al. 2014 [17]	-	-	-
Parent-level	Parkes et al. 2016 [34]	Goisis, Sacker, and Kelly 2016; Goisis, Martinson, and Sigle 2019; Walsh and Cullinan 2015 [31, 39, 42]	Wijlaars et al. 2011 [33]	-	-	Martinson, McLanahan, and Brooks-Gunn 2012 [37]
Child health	-	Goisis, Sacker, and Kelly 2016; Goisis, Martinson, and Sigle 2019; Walsh and Cullinan 2015 [31, 39, 42]	-	Noonan et al. 2016 [41]	-	Laverty et al. 2021; Noonan and Fairclough 2018 [30, 40]
Household-level	-	Walsh and Cullinan 2015 [42]	-	Mireku and Rodriguez 2020 [45]	-	Martinson, McLanahan, and Brooks-Gunn 2012 [37]
Geography	-	Walsh and Cullinan 2015 [42]	-	Cetateanu and Jones 2014; Noonan et al. 2016 [41, 46]	-	Schalkwijk et al. 2018 [38]
Ethnicity	-	Goisis, Martinson, and Sigle 2019 [31]	-	Strugnell et al. 2020 [44]	-	Lu, Pearce, and Li 2020; Martinson, McLanahan, and Brooks-Gunn 2012 [37, 43]
ACE	Straatmann et al. 2020 [26]	-	-	-	-	-
School-level		-	-	Townsend, Rutter, and Foster 2012 [32]	-	-
Child height		-	-	-	Samani-Radia and McCarthy 2011 [29]	-

Abbreviations: *ACE* Adverse Childhood Event**,** *SEP* Socioeconomic Position

### Assessment of study quality

A critical appraisal of each journal article was completed by researchers working independently in pairs. Disagreements were resolved by discussion. An adapted version of the Liverpool Quality Assessment Tool [23] and the Effective Public Health Practice Project Quality Assessment Tool [24] were used to measure study quality. In looking at baseline assessment, all (*n* = 23) studies were rated as having valid data collection tools, while 14 papers [17, 25, 26, 28, 30, 32, 34, 39–45] were considered strong for participant study completion (60–100%). Nine papers reported dropouts [17, 31, 32, 39–43, 46]. When analysing confounders, 13 studies [17, 25, 27, 28, 30–33, 38, 40, 42, 43, 46] had controlled for most, or some confounders. For all included studies, statistical methods were rated as appropriate for the study design. After several rounds of screening, all papers were scored highly for quality impact and considered applicable to the review. Overall scoring rated 10 studies of strong quality [25–27, 32, 34, 35, 38, 40, 42, 46], 12 of moderate quality [17, 28–31, 33, 36, 37, 39, 41, 43, 45], and one study of weak quality [44]. Table 2 reports the overall scoring for each individual study.

## Discussion

This review summarises research completed in Ireland and the UK in the last ten years, examining factors that mediate or attenuate SEP differentials in adiposity for children aged 18 and younger. Factors were examined according to definitions of SEP, and studies were appraised for study quality. A number of statistical methods were used in the studies, with some referencing specific strategies for assessing and comparing mediator models [56, 57]. Most studies used regression modelling. Some models reported results for aggregated mediating factors, making it impossible to assess the effect of individual factors in isolation. A critical appraisal of each journal article found the majority (22/23) to be of strong or moderate quality, and all included studies were applicable to the review question.

In Ireland and the UK, SEP differentials are evident from as early as three and nine months of age respectively [17, 33], are seen to persist during childhood, and to widen during adolescence [43]. There is a more pronounced differential reported at age 11 compared to age five [39], particularly when considered by ethnic group [31, 43, 44]. For the younger age groups, factors outside the home have less of an effect; for example, the availability of fast food, and other unhealthy foods outlets, did not mediate for younger children, but were found to mediate the SEP differential at age 10–11 years [46]. Place-based factors, such as safe environments and neighbourhoods were shown to be beneficial and were associated with increased rates of child physical activity, including active travel to school. Furthermore, the duration of time that children and young people spend in more deprived neighbourhoods and schools is associated with more harmful outcomes [32, 38, 40, 41, 45].

This review highlights that for some ethnic minority children, there are differences in the pattern of SEP differentials in adiposity outcome. The risk of OB or OW associated with low SEP is higher for White children compared to all “other ethnic backgrounds” [31]. A widening of the SEP differential in early childhood was evident when examined by most ethnic groups [31, 43, 44]; however, one study reported a negative association between BMI and SEP for children of Black origin [43]. Low SEP children with non-White native- and foreign-born mothers were found to be at lower risk of OW compared with low SEP children of White immigrant mothers [37]. Additionally, early life factors (including smoking during pregnancy and duration of breastfeeding) explained differences in the White group, but had no effect on the Black, Caribbean, and African groups [31]. The role of ethnicity in the association between SEP and childhood OW/OB outcome was not considered in either of the Ireland-based studies included in this review; it was only considered in UK-based research. This is of note as, according to the 2016 census, Ireland’s population had doubled since 1950, and one in ten of the population had been born outside Ireland. The census reported an increase in the proportion of Irish nationals identifying as other than ‘White Irish’, suggesting that research into what role ethnicity might play in the SEP/adiposity association would be beneficial when addressing this issue within the Irish context.

As with previous systematic reviews, this review found that early life factors were consistently identified as mediators in the SEP differentials in adiposity outcome. From three months through to 11 years of age, clear effects were seen for early life risk factors including maternal pre-pregnancy OW, breastfeeding duration, time of weaning, high birthweight, antenatal smoking, and alcohol consumption during pregnancy [17, 25, 31, 33, 35, 42]. One exception was found in a study employing family income as the SEP indicator; yet this same study repeated the analysis using education level as an SEP indicator and found early life factors (including high birthweight, maternal smoking during pregnancy, and not being breastfed) mediated the SEP differential [35]. This highlights the need for careful consideration of the measurement of SEP employed in the study design, and perhaps supports the use of multiple measures of SEP within a study.

A novel finding was the contribution of neighbourhood aesthetics and geographic-level deprivation to the SEP differentials for adiposity outcome. Furthermore, when SEP differentials (defined by family income) were examined by geographical-level deprivation, a u-shape association was seen with the family income effect dissipating for the moderately affluent areas but persisting for the most and least affluent areas. This suggests that moderately affluent areas may have ease of access to community amenities, while lower income families living in more affluent areas may be denied access to amenities by virtue of lack of public amenities or amenities being private or fee-based [45]. At age seven, higher BMI outcome for low SEP children in neighbourhoods with positive aesthetics were mediated by higher levels of physical activity, while evidence was found to indicate that individual-level and area-level SEP were independently related to adiposity outcomes. Surprisingly, in neighbourhoods with high aesthetics scores, higher physical activity reduced the SEP differential, suggesting that for this age, higher rates of OW and OB in the lower SEP group was not driven by physical inactivity [30, 41].

However, in comparison with early life factors, environment and ethnicity, the effect of child- and parent-level factors were less clear. Relatively few studies examined these factors in isolation. Child diet (including fruit intake, skipping breakfast, and sugar drink intake) and TV viewing contributed most to SEP differentials in younger children [17, 31, 34]. For children aged 14, the amount of screen time had the greatest effect on SEP differentials in OB outcome. While some studies reported high reductions in the SEP differentials when diet was considered [17, 34], others found less of an effect, reporting stronger effects for parent-level factors, such as allowing bedroom TV [42]. Indeed, some argued child diet should be considered an indirect effect of parenting [27].

As expected, the magnitude of an effect in the selected papers is related to the method used to classify adiposity (i.e. BMI, OW or OB). For instance, smoking during pregnancy, length of breastfeeding, and time of weaning are reported as having the greatest effect on the SEP association with OB, compared with OW [39]. In addition, different measures of disadvantage were found to capture different outcomes and were not interchangeable [42]. For example, high birthweight and maternal smoking in pregnancy mediated SEP differentials when SEP was measured using education, but not when using income [35]. This highlights that within this field, different measures of, and proxies for, adiposity (e.g. BMI, OB, OW) and SEP (e.g. level of maternal education, family income etc.) must be carefully considered when planning research and interpreting outcomes.

### Strengths and limitations

Previous research has shown that as a measure of SEP, level of maternal education is used most often in childhood OB literature and is considered to be more stable than other measures throughout the child’s upbringing [58, 59]. Notably, with increasing diversity in both Irish and UK populations, how maternal education is categorised must be considered, particularly in well-established datasets, such as the MCS and GUI. As an example, three of the four studies included in this review use the MCS categories of maternal education as an indicator of SEP. However, the MCS may miscategorise as many as 3% of the total sample or up to 10% of ethnic minority groups by providing an ‘overseas qualification only’ category which has no corresponding UK (or ISCED) education category [31]. It is therefore imperative that as new, more recent sweeps of important longitudinal datasets such as the MCS and GUI are interrogated, the measure of maternal education be adjusted for ethnicity. It is of note that Ireland has yet to publish research examining SEP differentials in terms of ethnicity, despite the ethnic composition of Ireland changing substantially in recent decades.

A strength of this review is that all studies used objectively measured child weight and height measurements. The majority (20/23 measurement points) were obtained using trained researchers and the balance using health professionals (n = 2) and school records (n = 1). Objective anthropometric measures protect against the divergence seen between clinical and parental measures, particularly at the extremes of the weight spectrum (with parents less likely to classify children as over-, or under-weight) [60, 61]. However, this objectivity did not extend to measurements of mediating factors (including diet and levels of physical activity) and indicators of SEP. The majority of studies (19/23) relied on questionnaires or interviews for data collection, which risks error due to participant embarrassment or recall bias.

One limitation to the study is the over-representation of certain datasets in each country: the Millennium Cohort Study is the source for 13/21 (62%) studies in the UK, while the Growing Up in Ireland dataset is the source for both studies (100%) based in Ireland. While both the Millennium Cohort Study and the Growing Up in Ireland study are very robust data sources that are well validated and representative of their respective population, the over-reliance on these datasets in examining childhood outcomes for both the UK and Ireland should be considered.

Finally, this review was designed to examine studies of children up to the age of 18 years. Despite the search terms, of the 23 studies in the review, the oldest children featured were aged 14 years. This lack of studies for the older adolescent cohort identifies a clear gap in the literature and prevents examination of the reach or progression of the effect of mediators. Targeting this shortfall is needed to fully inform future intervention strategies to address the SEP differentials in adiposity outcomes.

## Conclusion

Understanding what factors mediate the association between low SEP and adiposity is vital when planning interventions and considering policy development. This review identified several modifiable factors that confirm research undertaken in other OECD countries. Early life exposures, parenting practices, and school-level deprivation have been confirmed to be significantly associated with differences in adiposity outcomes. In addition, our review of the research for Ireland and the UK highlighted an important role for place-based factors (including neighbourhood safety and aesthetic characteristics). In keeping with previous OECD-wide reviews [19], our results are equivocal about the role of physical activity in the risk of OW and OB. This would suggest that more studies, ideally employing objective measures of activity, would be beneficial to further investigate this outcome.

Overall, our findings support many longitudinal studies showing the efficacy of targeted, early intervention programmes aimed at disadvantaged children and their families. Interventions designed to help mothers engage in beneficial health behaviours (e.g. maintaining a healthy weight, reducing smoking and/or drinking during pregnancy, increase rates and duration of breastfeeding), can improve outcomes for the child, mother and family, and can potentially address future risk of childhood OW and OB [25, 36, 62]. Indeed, the widening of SEP differentials seen as the child matures reinforces the need for these early, preventative interventions.

This review is particularly timely considering the dramatic widening of the deprivation gap evident in England’s National Child Measurement Programme of 2021. Since 2020, the SEP differential has almost doubled. Currently over 20% of five year old children living in the most deprived areas have OB, compared with under 8% of their more affluent peers [63]. In light of these figures, the relative paucity of research undertaken in Ireland and the UK examining mediators of SEP differentials for OB outcomes must be addressed urgently. Our findings suggest that while multi-country analyses provide an excellent overview, country- or area-specific research may produce more nuanced findings, which may better inform the development of policy responses and interventions.

## Supplementary Information

**Additional file 1.** 

## Data Availability

The dataset generated and/or analysed in this review can be accessed by replicating the search criteria outlined in Appendix 1 within the following databases: Ovid MEDLINE, Embase, Web of Science and EBSCOhost. Search results are also available from the corresponding author on reasonable request.
