# Supplementary material for: Mediators of socioeconomic differences in overweight and obesity among youth in Ireland and the UK (2011–2021): a systematic review
Source: BMC Public Health. 2022 Aug 20;22:1585. doi: 10.1186/s12889-022-14004-z (PMC9392918; doi:10.1186/s12889-022-14004-z)
Supplement: Supplementary file 1 — Additional file 1. [file 12889_2022_14004_MOESM1_ESM.docx]

**APPENDIX 1**

Full search strategy for Ovid MEDLINE presenting all limits applied.

|  | OVID MEDLINE  Ovid MEDLINE(R) and Epub Ahead of Print, In-Process, In-Data-Review & Other Non-Indexed Citations, Daily and Versions(R) 1946 to August 04, 2021 |  |
| --- | --- | --- |
| 1 | adiposity.mp. or exp Adiposity/ or *Body Weight/ or exp Obesity/ or obesity.mp. or overweight.mp. or exp Overweight/ or body mass index.mp. or exp Body Mass Index/ or BMI.mp. | 593500 |
| 2 | exp Socioeconomic Factors/ or exp Social Class/ or (socioeconomic* or socio$economic or education or occupation or income or SES or SEP or social class or social gradient or social status or social position or social place or social inequality*).mp. | 1485031 |
| 3 | (mediat* or attenuat* or indirect or decompos* or difference* or differential*).mp. | 5305799 |
| 4 | infant/ or infant.mp. or infants.mp. or exp Child/ or (child or children or childhood).mp. or exp Adolescent/ OR adolescent*.mp. or (youth or youths or teen*).mp. | 4177716 |
| 5 | (ireland or england or wales or scotland or uk or (united adj1 kingdom) or (great adj1 britain)).mp. | 511844 |
| 6 | 1 and 2 and 3 and 4 and 5 | 489 |
| 7 | Limit 6 to 2011 - 2021 | 322 |
